# Age and resilience amid COVID-19 pandemic adversity: the mediating roles of quality of life, spirituality, and depressive symptoms

**DOI:** 10.3389/fpsyg.2025.1576150

**Published:** 2025-08-12

**Authors:** Leonardo Gonçalves, Lorenzo Casagrande Reggiani, Josiane Maliuk, Gianfranco Rizzotto de Souza, Renato Gorga Bandeira de Mello, Bruno Perosa Carniel, Neusa Sica da Rocha

**Affiliations:** ^1^Clinical Research Center, Hospital De Clínicas De Porto Alegre, Porto Alegre, Brazil; ^2^Graduate Program in Psychiatry and Behavior Science, Universidade Federal Do Rio Grande Do Sul, Porto Alegre, Brazil; ^3^Innovations and Interventions in Quality of Life Research Group, Universidade Federal Do Rio Grande Do Sul, Porto Alegre, Brazil; ^4^Geriatrics Team, Internal Medicine Service, Hospital De Clínicas De Porto Alegre, Porto Alegre, Brazil; ^5^Psychiatry Service, Hospital De Clínicas De Porto Alegre, Porto Alegre, Brazil

**Keywords:** COVID-19, resilience, older adults, quality of life, spirituality

## Abstract

**Introduction:**

Resilience scores are usually higher among older adults, but the COVID-19 pandemic and the associated social isolation in this risk group necessitate a reevaluation of this characteristic.

**Objectives:**

To investigate the differences in resilience among young, middle-aged, and elderly individuals and to explore the mediating factors (quality of life, spirituality, social support, depressive symptoms) in the relationship between age and resilience.

**Methods:**

A cross-sectional study was conducted in April 2020 through online collection using the snowball method, enrolling 3,278 participants. They were divided into three age groups (18–36, 37–59, 60+), and resilience was assessed using the CD-RISC-10 scale. Mediation analyses examined the roles of depressive symptoms (PHQ-9), quality of life (EUROHIS-QOL-8), spirituality (WHOQOL-SRPB), and social support (MOS).

**Results:**

The sample comprised 1,207 young, 1,680 middle-aged, and 391 older adults. Resilience scores were significantly higher in the elderly population compared to middle-aged and young adults [F(2,3251) = 81.12; *p* = 0.001]. Quality of life (β = 0.23; *p* = 0.001) and spirituality (β = 0.28; *p* = 0.001) showed positive mediating effects, while depressive symptoms (β = −0.18; *p* = 0.001) had a negative effect. Social support did not show a statistically significant mediating effect.

**Conclusion:**

Older age was associated with higher resilience scores, even during COVID-19 pandemic. Spirituality and quality of life were identified as mediators of this relationship. These findings underscore the need for longitudinal research to confirm whether these factors predict resilience and to guide mental health interventions.

## Introduction

Resilience is a dynamic process of successful adaptation to adversity, from minor threats to significant trauma (American Psychological Association, 2024). Biological, environmental, social, and cultural factors are known to influence individual responses to adversity (Southwick et al., 2014; Gaffey et al., 2016). Old age is often associated with higher resilience compared to younger age groups (Gooding et al., 2012; Eshel et al., 2016; Na et al., 2022; Staneva et al., 2022). However, evidence supporting this difference is somewhat limited, as most studies compare young and older adults without including middle-aged individuals. This omission restricts the understanding of whether resilience increases progressively throughout life. Additionally, factors such as quality of life, spirituality, social support, and depressive symptoms have been shown to directly impact resilience (Nygren et al., 2005; Lamond et al., 2008; Hildon et al., 2010; Zhang et al., 2017; Schmitt et al., 2021; Londero and da Rocha, 2024). Their influence on the age-resilience relationship remains poorly understood until this moment.

The COVID-19 pandemic has highlighted the importance of resilience in mitigating the psychological impacts of prolonged stress and social isolation. Older adults, for instance, faced higher risks of morbidity and mortality (Banerjee and Rai, 2020; Jawaid, 2020), along with emotional and social challenges resulting from confinement (Griffiths et al., 2015; Shi et al., 2015). Despite these vulnerabilities, studies have paradoxically shown that older adults often report higher levels of resilience compared to younger individuals during crises prior to COVID-19 pandemic (Hansen and Slagsvold, 2012; Hikichi et al., 2016; Rafiey et al., 2016). This observation raises questions about the protective factors that contribute to resilience in different age groups and how these factors interact to shape adaptive capacities under stress.

Most studies on resilience focus on comparisons between younger and older adults, but often overlook middle-aged individuals. Gooding et al. (2012) examined resilience in young and older populations without including middle-aged adults as a distinct group. Similarly, Eshel et al. (2016) provided broader insights yet did not directly address whether middle age significantly influences resilience. Meanwhile, Na et al. (2022) stratified age into four groups, including middle age, but assessed resilience as a moderating variable in the relationship between age and mental distress, rather than as the main outcome.

Quality of life, encompassing both physical and psychological well-being, has been consistently linked to higher resilience (Hildon et al., 2010; Pardeller et al., 2020; Brinkhof et al., 2021). Individuals with better overall quality of life tend to cope more effectively with stress and adapt more readily to challenges (Hildon et al., 2010; MacLeod et al., 2016). Spirituality also plays an essential role in resilience, offering a sense of purpose and meaning that can strengthen individuals during periods of adversity (Nygren et al., 2005). On the other hand, depressive symptoms undermine resilience, making individuals more vulnerable to the psychological impacts of stress and adversity (Lamond et al., 2008; Schmitt et al., 2021; Londero and da Rocha, 2024). Finally, social support has been shown to provide both emotional and practical resources, acting as a crucial buffer against stress and promoting better mental health outcomes during crises (Gaffey et al., 2016; Southwick et al., 2016; Weitzel et al., 2021).

Despite these findings, little is known about how these factors interact to shape resilience across different age groups. Most existing research focuses on binary comparisons between young and older adults or is exclusively centered on older populations, often neglecting the transitional dynamics of resilience in middle-aged individuals (De Pue et al., 2021) and the role of psychosocial factors such as quality of life, spirituality, social support, and depressive symptoms. Investigating these interactions may help clarify the mechanisms underlying age-related differences in resilience and provide a more nuanced understanding of how protective and harmful factors influence resilience across the lifespan.

### Objectives and hypothesis

The primary objective of this study was to investigate whether there was a difference in resilience between age groups during the COVID-19 pandemic. We hypothesized that resilience levels increased according to age group. The secondary objective was to evaluate whether quality of life, social support, depressive symptoms, and spirituality mediate the relationship between age and resilience, by exploring how these mediators differ across age groups in the COVID-19 pandemic. We hypothesize that quality of life, social support and spirituality would exert a positive mediating effect, while depressive symptoms would act with a negative mediating effect.

## Materials and methods

### Data availability statement

Technical appendix, statistical code, and raw dataset available by contacting the corresponding author. This study was not pre-registered. Clinical trial number: not applicable.

### Study design and setting

This cross-sectional study, conducted during the early stages of the COVID-19 pandemic (April 14–23, 2020), utilized a structured online survey distributed via Google Forms. A snowball sampling method was employed to recruit participants. To comply with social isolation measures, the study was promoted through the virtual platforms of the Federal University of Rio Grande do Sul and social media, where the link to the research protocol was made available. Participants, all aged 18 years or older, provided informed consent before completing a 7-part questionnaire.

At the time of data collection (April 2020), Brazil was entering a critical phase of the pandemic, characterized by rapidly increasing infection rates, national lockdown recommendations, and significant uncertainty regarding the healthcare system’s capacity. Many participants were already experiencing restrictions on mobility, social isolation, and disruption to work and family routines. Since then, global COVID-19 cases had reached 676,609,955, with 6,881,955 reported deaths, as per data from the Johns Hopkins University Coronavirus Resource Center (Johns Hopkins University Coronavirus Resource Center, 2023), as of its final update in March 2023. In Brazil, by September 2024, 37,915,370 cases of infection and 713,205 deaths had been recorded, according to the Ministry of Health (BR) (2024).

### Instruments

The main research instrument was the Connor-Davidson resilience scale (CD-RISC-10), developed by Connor and Davidson (2003), and consists of 10 questions using a 5-point Likert scale. The scale has good psychometric properties, such as its convergent validity and predictive capacity, and was validated in Brazilian Portuguese by Solano et al. (2016). This protocol included demographic data, primarily age, but also gender, ethnicity, marital status, occupation, and education.

This protocol included demographic data, primarily age, but also gender, ethnicity, marital status, occupation, and education. The subsequent split of participants into three groups, which will be called Young Adults (YA) between 18 and 36 years old, Middle-aged Adults (MA) between 37 and 60 years old, and Older Adults (OA) over 60 years. The definition of older adults as people aged over 60 years was based on the concept established by the World Health Organization (WHO) for developing countries. For most developed countries, this age group includes people over 65. The other cut-off point was 35 years, as it is generally considered that an average adult is twice the minimum age of 18 years.

For mediation analysis, quality of life was assessed using the EUROHIS-QOL-8 questionnaire, validated in Brazilian Portuguese by Pires et al. (2018); spirituality using the WHOQOL-SRPB instrument, validated in Brazilian Portuguese by Fleck et al. (2000); social support by the MOS score, validated in Brazilian Portuguese by Griep et al. (2005), Preacher and Hayes (2004); and depressive symptoms using the PHQ-9 questionnaire, validated in Brazilian Portuguese by Santos et al. (2013).

### Statistical methods

The continuous variables were presented as means and standard deviations (SD). Categorical variables (gender, ethnicity, occupation, and education) were presented as percentages and compared using the chi-squared test. One-way analysis of variance (ANOVA) was used to compare age groups regarding resilience. Tukey’s *post-hoc* tests evaluated pairwise differences between groups. The mediation analysis was performed with the statistically significant factors obtained in the logistic regression using the bootstrapping method described by Preacher and Hayes (2004). The level of significance was set at *p* < 0.05. All analyses were performed using software SPSS version 21 (IBM Corporation, Armonk, USA).

### Ethics and consent to participate

This research followed the National Health Council (CNS) resolution 516/2016 determinations and the Declaration of Helsinki. It was assessed by the Research Ethics Committee of Hospital Gianfranco Rizzotto Renato Gorga Bandeira de Mello Souza Clínicas de Porto Alegre and approved by GPPG 2020/0141. Data were anonymized before constructing the database to be analyzed, not allowing participant identification. Informed consent was obtained from all individual participants included in this study.

## Results

**FIGURE 1:**
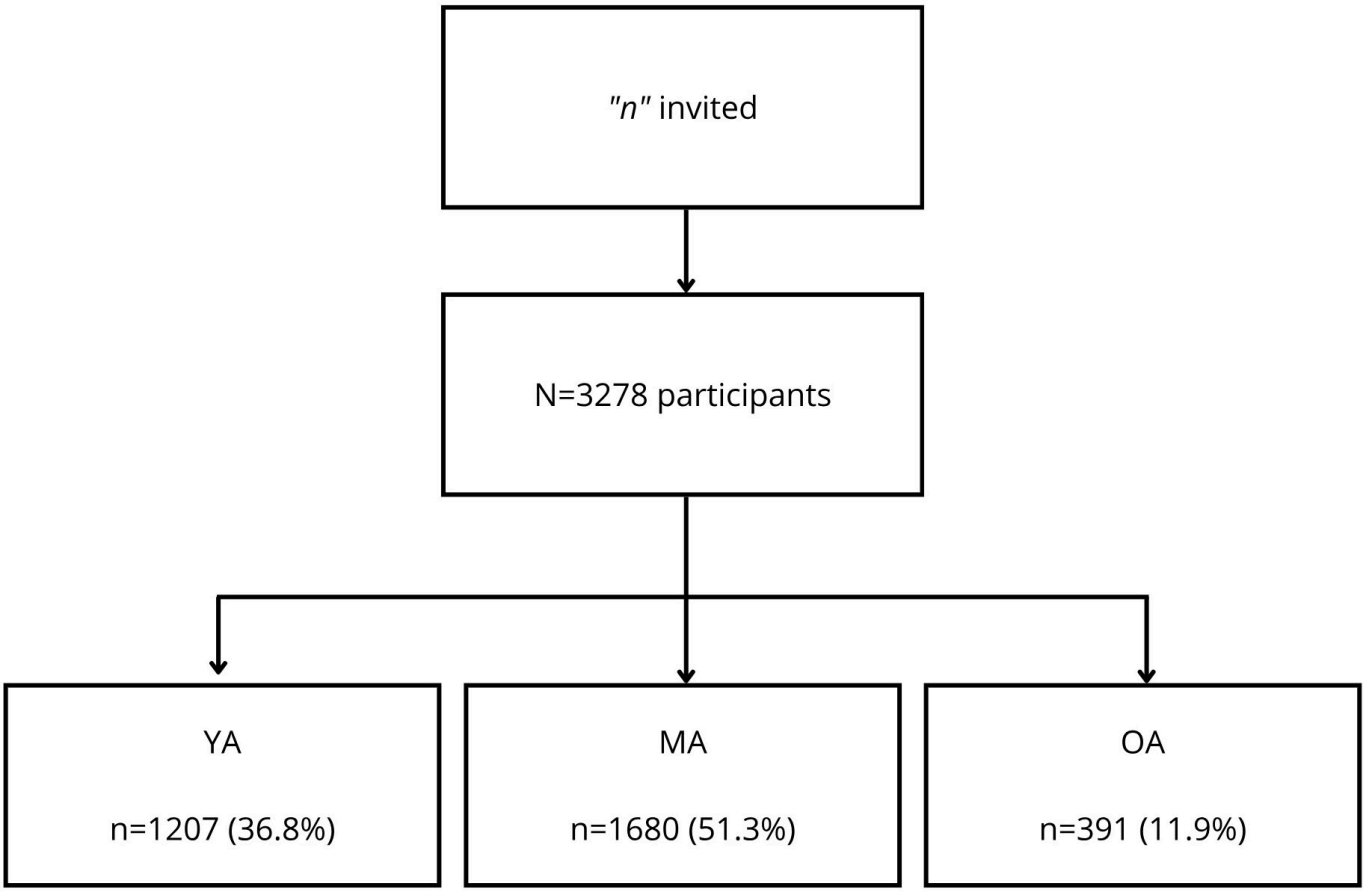
Flowchart of the sample of 3,278 adults during the COVID-19 pandemic. YA, Young adults; MA, Middle-aged adults; and OA, Older adults.

The flowchart (Figure 1) provides us with an overview of the result of the inclusion of participants and age group split. In total, 3278 participants were obtained, divided into three groups, which will be called Young Adults (YA) between 18 and 36 years old, Middle-aged Adults (MA) between 37 and 60 years old, and Older Adults (OA) over 60 years. We obtained a total of 1207 (36.8%) in the YA group, 1680 (51.3%) in the MA group and 391 (11.9%) individuals in the EA group.

Table 1 shows the sociodemographic characteristics of each group. The mean age in the YA group was 28.4 years (SD ± 5.03); in the MA group, it was 47.1 years (SD ± 6.72); and in the OA group, it was 65.1 years (SD ± 4.62). In the three groups, women were predominant (54%–87%) of white ethnicity (88%–95.3%) and who had at least completed high school (63.5%–84.3%). Regarding marital status, there was a predominance of married individuals in the YA (50.9%) and MA (72.1%) groups and Widow/widower individuals in the OA group (68.4%). As for occupation, there was a predominance of individuals with paid employment in the YA (64.5%) and MA (80.2%) groups, while in the OA group, retired people were predominant due to age (57.4%). Responders were mainly health care professionals (31.8%) and young or middle-aged adults; only 11.9% were categorized as older adults. Out of all participants, 0.4% stated being a suspected case of COVID-19, and 16.5% reported having some chronic disease, of which middle-aged adults presented the most (55.7%).

**TABLE 1:** Sociodemographic characteristics of the sample of 3,278 adults.

Characteristic	Young adult (18–36 years)	Middle-aged adult (37–59 years)	Older adults (≥60 years)	Total (*N* = 3278)	χ 2/F	*P*
**Age group, *n* (%)**	1207 (36.8)	1680 (51.3)	391 (11.9)			
**Age, mean (±SD)**	28.43 (±5.03)	47.08 (±6.72)	65.15 (±4.62)	42.37 (±13.41)		
**Sex, *n* (%)**					9.667	0.008
Male	290 (41)	326 (46)	92 (13)	708 (21.6)		
**Ethnicity, *n* (%)**					27.139	<0.001
White	1055 (88)	1544 (92.5)	368 (95.3)	2967 (91.2)		
Non-White	144 (12)	125 (7.5)	18 (4.7)	287 (8.8)		
**Marital status, *n* (%)**					743.843	<0.001
Single	566 (47.5)	234 (14.1)	33 (4)	833 (8.7)		
Married or cohabiting	607 (50.9)	1194 (72.1)	220 (10.9)	2031 (58)		
Separated or divorced	18 (1.5)	211 (12.7)	87 (27.5)	318 (23)		
Widow/widower	1 (0.1)	17 (1.0)	39 (68.4)	58 (10.3)		
**Occupation, *n* (%)**					1537.470	<0.001
Retired due to disability	1 (0.1)	11 (0.7)	8 (2.1)	20 (0.6)		
Retired due to age	0 (0)	112 (6.8)	217 (57.4)	332 (10.1)		
With paid occupation	762 (64.5)	1316 (80.2)	134 (35.4)	2219 (67.9)		
Homemaker	32 (2.7)	78 (4.8)	11 (2.9)	122 (3.7)		
On sick leave	3 (0.3)	16 (1.0)	2 (0.5)	21 (0.6)		
Student	329 (27.9)	35 (2.1)	0 (0)	365 (11.2)		
Without occupation (not retired)	54 (4.6)	73 (4.4)	6 (1.6)	134 (4.1)		
**Education, *n* (%)**					300.114	<0.001
Incomplete primary education	0 (0)	6 (0.4)	3 (0.8)	9 (0.3)		
Complete primary education	3 (0.3)	5 (0.3)	1 (0.3)	9 (0.3)		
Incomplete secondary education	7 (0.6)	8 (0.5)	7 (1.8)	22 (0.7)		
Complete secondary education	85 (7.1)	90 (5.4)	30 (7.8)	205 (6.3)		
Incomplete higher education	343 (28.6)	153 (9.2)	26 (6.7)	522 (16)		
Complete higher education	298 (24.8)	356 (21.3)	118 (30.6)	772 (23.7)		
Graduate education	464 (38.7)	1052 (63)	201 (52.1)	1717 (52.7)		
**Resilience, mean (SD)**	25.06 (6.77)	27.22 (6.41)	29.56 (5.92)	26.72 (6.66)	5.20	<0.0001
**Health care professional, *n* (%)**	434 (35.9)	518 (30.8)	90 (23)	1043 (31.8)	22.250	<0.001
**Suspected COVID-19 case, *n* (%)**	3 (0.25)	11 (0.65)	0	14 (0.42)	4.630	<0.001
**Chronic disease, *n* (%)**	141 (11.7)	301 (18)	98 (25)	540 (16.5)	43.653	<0.001

SD, Standard deviation.

**FIGURE 2:**
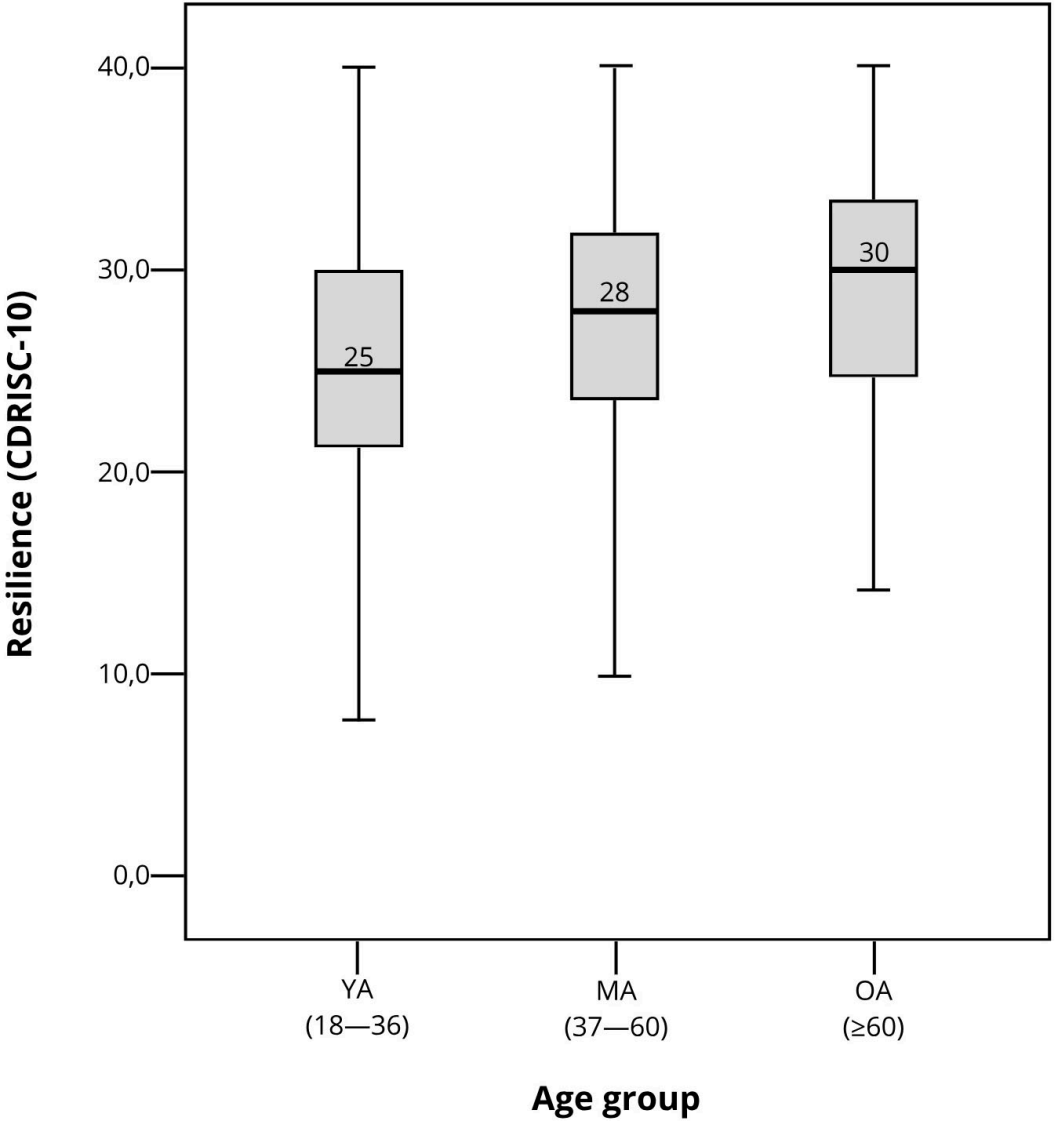
Box plot of resilience levels by age group during the COVID-19 pandemic. YA, Young adults; MA, Middle-aged adults; and OA, Older adults.

When comparing resilience levels (Figure 2), there was a significant difference between age groups [F (2,3251) = 81.12; *p* = 0.001]. A Tukey’s *post-hoc* test showed significant differences both between young and middle-aged adults [ΔM = −2.16; 95% confidence interval (CI), −2.74 to −1.58; *p* = 0.001)] and between middle-aged and older adults (ΔM = −2.34; 95% CI, −3.20 to −1.48; *p* = 0.001). Subgroup analyses showed that older adults aged over 70 years presented higher mean resilience than older adults below this age. Still, this difference was not statistically significant, possibly due to the small sample size within this age range.

The univariate analysis included the following factors associated with resilience: age, gender, quality of life (EUROHIS-QOL), spirituality (WHO-QOL SRPB), social support (MOS), diagnosis of depression (PHQ-9 > 5), and depressive symptoms (PHQ-9) (Table 2). Quality of life (ρ = 0.446; *p* = 0.001) and spirituality (ρ = 0.417; *p* = 0.001) had the greatest positive associations, while depressive symptoms presented a negative association (ρ = –0.506; *p* = 0.001).

**TABLE 2:** Univariate analysis of factors associated with resilience during the COVID-19 pandemic.

Factors	Category	Spearman’s Ro	*t*-test	*P*
Age		0.257		<0.001
Sex	Male		26.37 (6.66)	<0.001
	Female		28 (6.43)	
Spirituality^1^		0.449		<0.001
Social support^2^		0.254		<0.001
Quality of life^3^		0.463		<0.001
PHQ-9		−0.506		
Depression diagnosis	Yes		28.11 (5.96)	<0.001
	No		22.19 (6.79)	

CI, Confidence interval;

^1^Evaluated through the abbreviated World Health Organization Quality of Life - Spirituality, Religiousness, and Personal Beliefs (WHOQOL SRPB BREF);

^2^Evaluated through Social Support Questionnaire – Medical Outcomes Study (MOS);

^3^Evaluated through EUROHIS-Quality of Life (QOL) 8-item index.

In the stepwise multivariate analysis (Table 3), the depression diagnosis (PHQ-9 > 5) predictor presented no significant association (*p* > 0.2) and was excluded from the final model. The complete model explained 34.3% (*B* = 34.3, *p* = 0.0001) of the variations in resilience, corresponding to a moderate effect size. The most important predictor of higher resilience scores was spirituality (β = 0.28; *p* = 0.001). A depression diagnosis was a negative predictor of resilience levels (β = −0.18; *p* = 0.001).

**TABLE 3:** Multivariate analysis of resilience predictors during the COVID-19 pandemic.

Predictor	B (95% CI)	Standardized β	*P*
Age	0.71 (0.41–1.01)	0.07	<0.001
Sex (ref. = female)	1.63 (1.16–2.09)	0.1	<0.001
Spirituality^1^	0.56 (0.49–0.63)	0.26	<0.001
Social support^2^	0.48 (0.24–0.71)	0.06	<0.001
Quality of life^3^	1.91 (1.51–2.31)	0.17	<0.001
Depression symptoms^4^	−0.27 (−0.31 to 0.23)	−0.25	<0.001
Health care professional	0.59 (0.19–0.99)	0.04	<0.04

CI, Confidence interval;

^1^Evaluated through the abbreviated World Health Organization Quality of Life - Spirituality, Religiousness, and Personal Beliefs (WHOQOL SRPB BREF);

^2^Evaluated through Social Support Questionnaire – Medical Outcomes Study (MOS);

^3^Evaluated through EUROHIS-Quality of Life (QOL) 8-item index;

^4^Evaluated through Patient Health Questionnaire-9 (PHQ-9).

**FIGURE 3:**
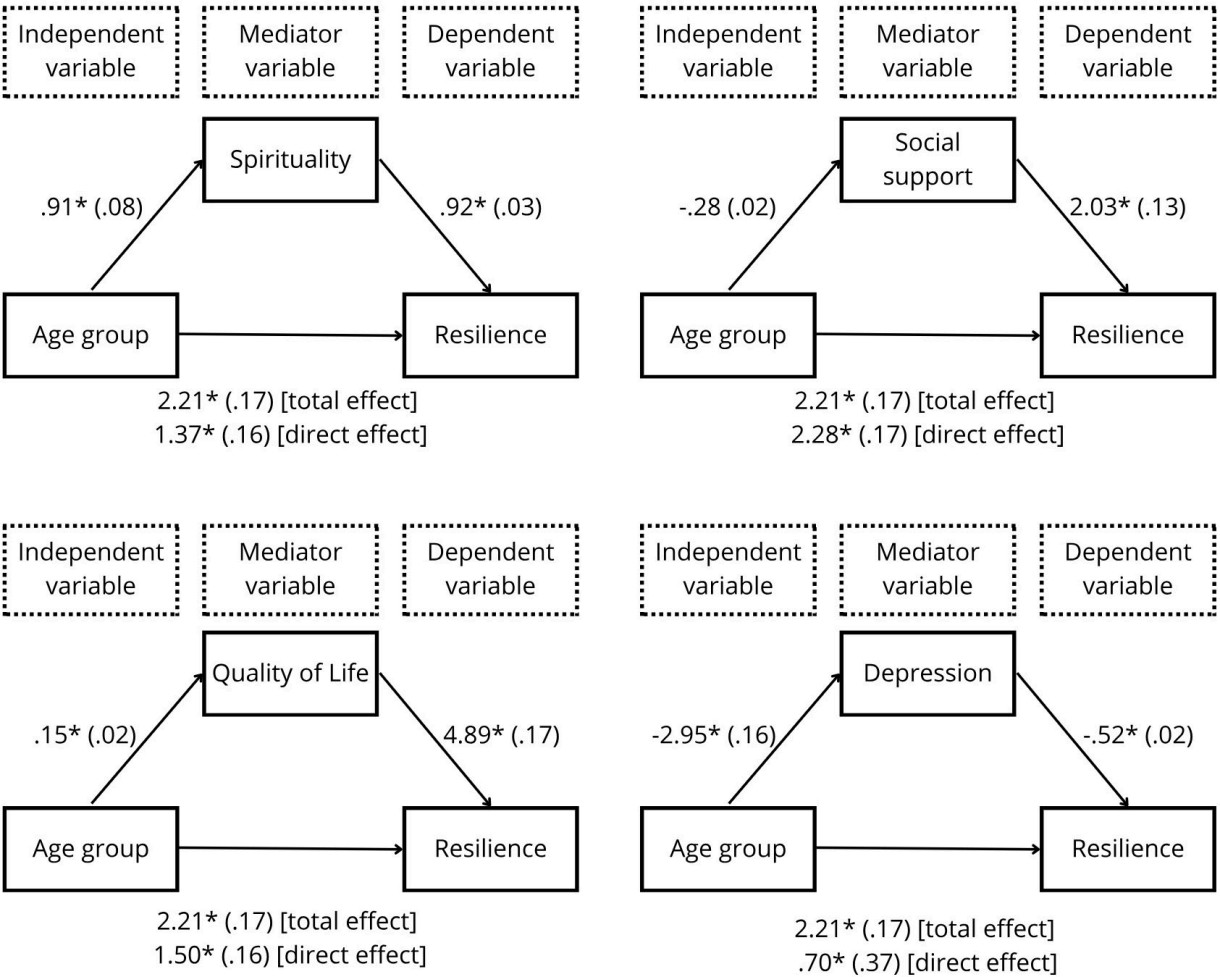
Age group model as a predictor of resilience, mediated by spirituality, quality of life, social support, and depressive symptoms. All coefficients represent unstandardized beta coefficients (standard errors in parentheses) *p = 0.001.

The age group model for resilience used spirituality, quality of life, social support, and depressive symptoms as mediators. Figure 3 shows that the mediation effect was statistically significant for spirituality [*b* = 0.83; BCaCI 95% (0.68, 1.0); R^2^ = 0.22; *p* = 0.001], quality of life [*b* = 0.73; BCaCI 95% (0.57, 0.89); R^2^ = 0.23; *p* = 0.001], and depressive symptoms [*b* = 1.52; BCaCI 95% (1.34, 1.72); R^2^ = 0.26; *p* = 0.001).

## Discussion

This study is the first to identify significant differences in resilience among young, middle-aged, and older adults, and to analyze the mediating effects of quality of life, spirituality, depressive symptoms, and social support in the age–resilience relationship during the COVID-19 pandemic. Our findings confirm that resilience increases with age, a pattern significantly mediated by spirituality, quality of life, and depressive symptoms. However, social support did not emerge as a significant mediator in this context.

The primary result showed that resilience increased by approximately 2 points between age groups, with the older population (>60 years) being the most resilient. In the subgroup analysis of older people, those aged over 70 years still presented higher mean resilience by 1 point, although this difference was not statistically significant, likely due to the small sample size in this age group. This finding suggests the existence of a subpopulation known as the oldest-old, as proposed in previous studies (Shen and Zeng, 2010; Zeng and Shen, 2010). Studies attributing higher resilience with age highlight factors such as expanded coping strategies (Fontes and Neri, 2019), emotional stability, and better social resources (Nygren et al., 2005; Fontes and Neri, 2015; Hayman et al., 2017). However, other studies emphasize challenges such as the prevalence of chronic diseases and social isolation (Beutel et al., 2009, 2010), which can reduce resilience when stressors become overwhelming (Crane et al., 2019).

The mediation analysis revealed notable findings. Spirituality and quality of life emerged as strong positive mediators, underscoring their clinical importance in supporting resilience, particularly among older adults. In contrast, depressive symptoms were negatively associated with resilience, reinforcing the need for early detection and treatment, especially during crises. The overall increase in protective factors with age suggests a generally healthier older population, which may reflect a socioeconomic bias in the sample. Notably, the mediation models did not include sex or health care professional status as covariates, as this was not among the objectives of the study. These variables, however, were included in the multivariate regression analyses (Table 3).

Although social support showed a direct positive association with resilience, its contribution to the mediation model was not statistically significant. Previous studies have shown a positive association (Hayman et al., 2017) or no association (Silva Júnior et al., 2019) between these constructs. The impact of social support may have been minimized by restricted access to resources, such as family, friends, and religious or social groups, during the early quarantine period when the sample was collected. The instrument used (MOS score) did not specifically consider the alternative forms of contact, such as social networks and videoconferencing, which became crucial during this time. Additionally, social support is not universally effective, as its benefits depend on the type of support provided and how well it matches the individual’s needs (Southwick et al., 2016).

This study contributes to consolidating the hypothesis that levels of resilience are higher among older individuals, who, despite being the focus of health concern during the COVID-19 pandemic, were possibly not the most affected in terms of quality of life. Research has shown that children and adolescents experienced significant decline in quality of life during the pandemic, influenced by reduced social interaction and increased unpredictability, which are linked to higher rates of depression (Araujo et al., 2024; Ye et al., 2024). Resilience has been associated with better mental health outcomes in these younger groups, including reduced anxiety and depression when paired with strong social support and adaptive coping mechanisms (Jones et al., 2021). Similarly, among young adults, higher resilience during the pandemic was linked to more positive coping with traumatic experiences (Xu et al., 2024). These findings highlights the clinical importance of assessing resilience across all age groups, particularly younger individuals, to better inform mental health interventions.

This study has several strengths. By analyzing the mediating effects of psychosocial factors–such as quality of life, spirituality, depressive symptoms, and social support–on the age-resilience relationship, it provides valuable insights into resilience dynamics across different age groups. The inclusion of three distinct age groups goes beyond traditional young-versus-older comparisons, offering a broader understanding of how resilience evolves throughout life. The large sample size (>3,000 participants) strengthens the robustness of the findings, while the study’s focus on resilience during the COVID-19 pandemic adds relevance for understanding psychological responses during crises.

Despite the strengths of this study, some limitations should be noted. First, its cross-sectional design limits the ability to establish causal relationships. Longitudinal, prospective studies are needed to clarify how age, resilience, and psychosocial variables interact over time. Second, the use of self-report measures may have introduced social desirability bias, a common limitation, especially in studies conducted during the first months of the COVID-19 pandemic. Third, the online snowball sampling likely led to selection bias. Most participants were predominantly White (91.2%), while national census data indicate that only 43.5% of Brazilians identify as White (Federal Government (BR), 2023). Other ethnic groups, such as Black, Indigenous, and Asian individuals, were underrepresented. In addition, more than half of the participants held a graduate degree, and 31.8% were healthcare professionals, mostly among YA (35.9%) and MA adults (30.8%). These patterns suggest an overrepresentation of individuals with higher education, socioeconomic status, and digital access, as noted in similar studies (Li et al., 2020; Zhang and Ma, 2020), which limits the generalizability of the findings. Finally, although sex and health care professional status were included as control variables in the multivariate regression models (Table 3), they were not included in the mediation models, as this was not among the study’s objectives. Future research may benefit from employing more advanced statistical approaches, such as structural equation modeling, to account for demographic covariates in mediation analyses. Additionally, more inclusive recruitment strategies may help improve representativeness and external validity.

## Conclusion

This study aimed to evaluate the associations of resilience in the COVID-19 outbreak among three age groups and its mediators, such as quality of life, spirituality, depressive symptoms, and social support. Resilience was found to be higher among older individuals, with quality of life and spirituality serving as significant positive mediators. Notably, depressive symptoms negatively contributed, whereas social support did not present a significant mediating effect.

## Data Availability

The raw data supporting the conclusions of this article will be made available by the authors, without undue reservation.
